# Moderating Effects of Intercultural Social Efficacy and the Role of Language in the Context of Coping Strategies in Study Abroad Depression

**DOI:** 10.3390/ijerph19042409

**Published:** 2022-02-19

**Authors:** Yueh-Luen Hu, Amy Roberts, Gregory S. Ching, Pei-Ching Chao

**Affiliations:** 1 Department of Education, National Chengchi University, Taipei City 116011, Taiwan; joyhu@nccu.edu.tw (Y.-L.H.); 99152513@nccu.edu.tw (P.-C.C.); 2 School of Teacher Education, University of Wyoming, Laramie, WY 82071, USA; aroberts@uwyo.edu; 3 Graduate Institute of Educational Leadership and Development, Fu Jen Catholic University, New Taipei City 24205, Taiwan; 4 Research and Development Center for Physical Education, Health, and Information Technology, Fu Jen Catholic University, New Taipei City 24205, Taiwan

**Keywords:** study abroad, Taiwan, intercultural social efficacy, coping strategies, depression

## Abstract

This study examined the relationship between intercultural social efficacy (ISE), coping strategies, Mandarin Chinese and English language proficiency, and depression. In total, 1870 foreign students in Taiwan participated in the study. Study results indicated that aspects of background demographics may influence depression levels. Participants with an immigrant background and those who are older are more likely to suffer from depression; however, gender and length of stay do not seem to affect depression. The moderating effects of Mandarin Chinese and English language proficiency and ISE on the relationship between coping strategies and depression were examined. Based on the results, the moderating role of Mandarin Chinese and English language proficiency was not supported, suggesting that coping strategies are independent of linguistic proficiency. However, knowing both languages is an important factor in reducing the stress of studying abroad. Additionally, the results confirmed the moderating effects of ISE, suggesting that a higher level of social effectiveness reinforces the negative association between coping strategies and depression. Students who are better able to interact with other cultures may be able to develop effective coping strategies. Moreover, this study found that although most of the study abroad students were not depressed, early intervention and prevention measures could help alleviate possible mental health crises.

## 1. Introduction

Renewed interest in study opportunities abroad has recently increased as the anticipation of a post-COVID-19 scenario grows. In addition, with the gradual return to face-to-face teaching, there is an increased focus on the provision of mental health support services, as indicated by 70% of potential study abroad applicants [1]. In fact, even before the pandemic, researchers already recognized that students studying abroad were prone to depression [2,3]. A recent analysis of study abroad articles published over the past 40 years identified growing concerns about students’ mental health, particularly their relationship to language, including the different social and psychological difficulties [4]. This brought attention to the fact that while studying abroad, students may be prone to mental illnesses such as depression.

As part of discussing the subject of study-abroad-related depression, it is also pertinent to highlight the importance of coping strategies. Importantly, as international students have become increasingly significant contributors to the college population, their mental health needs in particular need to be taken into account [5]. Study abroad coping strategies have also received considerable attention in recent years. In addition to concentrating on the health and lifestyle of students [6,7], this emphasis has evolved with the inclusion of the social aspect of studying abroad [8]. More important, it is also necessary to consider the impact of the host country’s language on academic and lifestyle challenges, which is an important agent within the sociocultural adaptation process [9,10,11].

Prior to the COVID-19 pandemic, there were approximately 130,000 foreign students studying in Taiwan [12]. A significant contribution to this development is the New Southbound Talent Development Program [13]. This program has resulted in a substantial increase in students from Southeast Asian countries studying in Taiwan, increasing from 32,000 students in 2016 to approaching 60,000 students in total during the 2019 academic year; a growth rate of around 85% [14]. Nevertheless, recent reports have pointed out that despite the pandemic many students from the region are quite eager to study in Taiwan [15]. In light of these facts, a deeper understanding of the subject of depression associated with studying abroad in Taiwan might be of interest, which is currently quite limited. Previous studies in Taiwan have focused on international students’ depression as a result of their internet usage habits [16], service quality [6], and cultural strategies [3], without providing an adequate examination of the role of language and sociocultural adaptation. 

As a theoretical framework for the study, Yu et al.’s [17] sociocultural adaptation is a dynamic process that includes language skills, academic efficacy, contact with locals, social supports, perceived discrimination, and psychological adjustment (p. 3). According to them, knowledge and skills in both the host language and in English play a vital role in the adaptation process. Furthermore, by building language and communication skills in the host language, many students would be able to have a positive study abroad experience [10,18]. Moreover, social interactions and engagements that enhance social support, whether academic (in the classroom) or community-based, are effective means of promoting the sociocultural adaptation process [8,17,19]. In fact, a number of studies have shown that the lack of interaction or the feeling of detachment due to the lack of social connection is actually a leading cause of study abroad depression [20].

Within the psychological adjustment of the sociocultural adaptation process, individuals may experience various stressful aspects of the acculturation process that stem from practical or environmental factors such as adapting to new foods, living arrangements, transportation systems, etc. [17]. These adaptations to cultural differences are actually the stressful aspects of studying abroad [21], and may lead to feelings of distress, loneliness or homesickness, that could result in depression [22]. Typically, such phenomena that are common among university students worldwide fall within an intermediate stage between being healthy and having depression: a so-called subthreshold depression. [23]. Subthreshold depression may worsen or recover with appropriate interventions. Consequently, psychologically adapting to these various stressors is crucial for the sociocultural adaptation process and can be considered as an intervention [24]. Importantly, the extent of psychological adaptation actually constitutes an integral element for determining the effectiveness of intercultural adjustment [25]. In essence, psychological adjustment operates within a stress and coping framework, which can be alleviated by utilizing various coping strategies [26].

**Figure 1:**
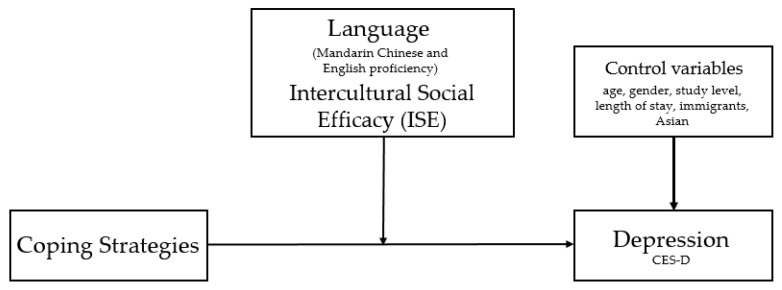
Theoretical framework of the study.

As a result of this framework, the present study aims to test the hypothesis that language (Mandarin Chinese; host country language and English) and intercultural social efficacy (ISE) act as moderators within the relationship between study abroad coping strategies and depression (see Figure 1). In addition, background demographic data were used as control variables. For example, it has long been established that depression is highly dependent on the individual’s age and gender [27,28]. Similarly, the length of stay has been observed to be associated with depression during study abroad [20,29]. In addition, the participants were also asked whether their families were immigrants; wherein their family has previously resided in another country. This information was collected with the awareness that students who were immigrants themselves have a pre-existing experience of being exposed to different cultures (and languages) [30,31], making them more receptive to intercultural interactions.

In addition to testing the proposed moderating effects of language (Mandarin Chinese and English) and ISE, two other goals are also included:

1. To validate an instrument used to measure study abroad students’ coping strategies and ISE;
2. To determine the role of coping strategies, ISE and Mandarin Chinese and English language proficiency in predicting depression.

## 2. Materials and Methods

### 2.1. Study Design and Participants

The present study was designed as a cross-sectional study in which this type of data collection can be used to examine information with different variables in order to obtain information about a population over a specific period of time [32]. The data used in this study are part of a multi-year project aimed at understanding the model of acculturation among international students in Taiwan. In addition to the consent form, participants were informed that the survey would not only collect information regarding depression, but would also stimulate reflection on their own experiences and perceptions concerning their language ability in both Mandarin Chinese and English in Taiwan.

Based on the 2016 Ministry of Education statistics, there were approximately 112,000 international students studying in Taiwan during that academic school year. The Sampsize program [33] was used to calculate the minimum sample size needed: 383 participants with a 5% margin of error and 95% confidence level. The inclusion criteria called for college or university students who were not Taiwan nationals and who were over 18 years of age. In total, 1958 international students participated in the semester-long data collection process using an online survey. Participants were also free to choose whether to use an English or Mandarin Chinese version of the survey. After removing non-participation, data from the remaining 1870 participants were screened for outliers and missing items that accounted for less than 10% of the entire dataset, which were later imputed using the expectation maximization algorithm [34]. Among the participants, 925 were female and 945 were male students. The average age was 26 years old, and the average length of stay was approximately 15 months. Cronbach’s [35] alpha reliability of the survey was computed as 0.84, denoting acceptable internal consistency [36]. The protocol of the study was reviewed and approved by the Fu Jen Catholic University Institutional Review Board.

### 2.2. Measures

As noted within the framework, participant background included information regarding age, gender, study level, length of stay (in months), whether the families were immigrants or not (answerable with a yes or no), and whether they were from Asia or not (answerable with a yes or no). The level of study was included because graduate study presents increased challenges in terms of research, raising funds, and securing future career opportunities; therefore, it was believed that a higher level of education would be more onerous [37]. Moreover, Asian students generally experience more academic pressure, resulting in greater stress than students from Europe or North America [38,39].

To measure the degree of depression in the participants, the 20 items Center for Epidemiologic Studies Depression Scale (CES-D) was administered [40]. The CES-D is a widely used tool for assessing the psychological distress of individuals. A further advantage of the CES-D is that it can be used to analyze the prevalence of subthreshold depression among students [23]. In a recent study, the CES-D was used to understand COVID-19-related depression among international students in Germany [41]. Furthermore, even before the pandemic began, the CES-D was used in Japan [42], the United States [43], and Taiwan [6,16] to assess depression levels among international students, to name a few. As a general rule, depression scores greater than or equal to 16 are considered depressed [40]. The Cronbach’s alpha reliability of the CES-D was computed as 0.84, denoting acceptable internal consistency. 

In terms of language proficiency, participants were asked to rate their self-perceived proficiency levels of Mandarin Chinese and English. Among the ratings were beginner, low intermediate, intermediate, high intermediate, and advanced. Depending on the field of study, international students in Taiwan are required to take either a proficiency test in English or Mandarin Chinese [44,45]. Although self-reported language proficiency is sometimes accompanied by an array of limitations, it can nonetheless provide an easy and reasonably accurate indicator of how students actually perceive their language competencies [46].

In terms of coping strategies, as indicated by Yu et al.’s [17] framework for psychological adjustment, a large part of the sociocultural adaptation process is devoted to the strategies employed in coping with perceived stressors. Coping strategies should not only encompass the concepts of health and lifestyle [7]; several other concepts have also been considered, including self-actualization [47], social support [48], and in-class rapport [49]. A total of 35 items with seven subscales were proposed, as follows:

- Social support—comprising eight items that describe the presence of someone who provides support or comfort during times of joy or sorrow. Sample items are “there is a special person who is always there to assist me” and “there are some friends with whom I can share both joys and sorrows”;
- Self-actualization—comprising six items that describe the realization of one’s talents and potentials. Sample items are “I feel that I am growing and changing in a positive direction” and “I look forward to the future”;
- Classroom rapport—comprising five items that describe the relationship the students have with their peers. Sample items are “I have a harmonious relationship with my classmates” and “I am comfortable interacting with my classmates”;
- Family support—comprising five items that describe the relationship the students have with their family. Sample items are “I get the emotional help and support I need from my family” and “I get in touch with my family all the time”;
- Health responsibility—comprising four items that describe how students are responsible and aware of their physical and mental well-being. Sample items are “Observe my body at least once a month for signs of changes” and “seek information from health professionals (doctor and/or counselor) about how to take good care of myself”;
- Daily routine—comprising four items that describe how students maintain a healthy lifestyle. Sample items are “I engage in recreational physical activities” and “I exercise regularly”;
- Self-relaxation—comprising three items that describe how students recognize the importance of slowing down and enjoying life. Sample items are “I take some time to relax each day” and “I am aware of my limitations”.

Data were collected using a five-point Likert-type scale [50] perceived level of agreement, wherein 1 = strongly disagree and 5 = strongly agree. Further information on construct validity and reliability can be found in Section 3.

For the ISE, a total of 18 items were adapted and revised from the Social Self-Efficacy Scale for Students [51]. Within the ISE, a range of social interaction situations, including those that may be particularly challenging for newcomers from cultures other than their own, were collected. The ISE consists of four subscales, namely:

- Absence of social difficulty—comprising eight items that describe how students interact with their peers and faculty. Sample items are “I find it easy to hold a conversation with most people” and “I am able to talk to university staff”;
- Social confidence—comprising five items that describe how confident the students are in dealing with others. Sample items are “I feel confident asking a question during class” and “I feel confident talking to my teachers”’
- Showing interest—comprising three items that describe the manner in which students are interested in social interaction. Sample items are “I enjoy activities that most Taiwanese students enjoy” and “I have common topics for conversation with Taiwanese students”;
- Friendship initiative—comprising two items that describe the students’ enthusiasm for social engagement. Sample items are “If I see someone I would like to meet, I go to that person instead of waiting for him/her to come to me” and “When I’m trying to become friends with someone who seems uninterested at first, I don’t give up easily”.

Data were collected using a five-point Likert-type scale perceived level of agreement, wherein 1 = strongly disagree to 5 = strongly agree. Further information on construct validity and reliability can also be found in Section 3.

## 3. Results

### 3.1. Background Demographics and Depression

The characteristics of the participants and the distribution of depression are shown in Table 1. Group comparisons were performed using independent samples *t*-test and ANOVAs were computed with SPSS version 26.0 (IBM, Armonk, NY, USA), on lease agreement from Hearne software. No significant differences were found with regard to gender, study level, or length of stay. Significant differences in depression scores were found among participants’ ages with *F* (2, 1867) = 10.73, *p* < 0.001, whereas post hoc analysis showed that both 26- to 35-year-olds (G2; M = 13.30, SD = 8.16) and those aged over 35 (G3; M = 13.54, SD = 7.19) were significantly higher than participants aged 25 years old and under (G1; M = 11.44, SD = 8.95). Significant differences were found among participants who were immigrants with *t* (1868) = 3.12, *p* = 0.002, wherein participants with a migrant background (G2; M = 13.47, SD = 8.52) showed significantly higher depression scores than those without a migrant background (G1; M = 11.83, SD = 8.65). Significant differences were also found among participants who were Asians with *t* (1868) = 2.21, *p* = 0.027, wherein participants from Asia (G2; M = 12.36, SD = 8.82) had significantly higher depression scores than those who were not from Asia (G1; M = 11.39, SD = 8.12).

For Mandarin Chinese proficiency, significant differences in depression scores were found among participants with *F* (4, 1865) = 6.17, *p* < 0.001, whereas a post hoc analysis showed that participants who rated themselves as high-intermediate (G4; M = 13.63, SD = 8.23) were significantly more depressed than those who rated themselves as advanced (G5; M = 11.30, SD = 8.86). Lastly, for English proficiency, significant differences in depression scores were also found among participants with *F* (4, 1865) = 12.23, *p* < 0.001, whereas post hoc analysis showed that participants who self-rated as beginners (G1; M = 19.00, SD = 7.89) were actually depressed (scores above 16) and had significantly higher depression scores than the participants who self-rated as intermediate (G3; M = 12.66, SD = 7.56), high-intermediate (G4; M = 11.34, SD = 8.58), and advanced (G5; M = 12.29, SD = 8.79). In addition, participants who rated themselves as low-intermediates (G2; M = 17.71, SD = 8.04) were also depressed and had significantly higher depression scores than the participants who rated themselves as intermediate (G3), high-intermediate (G4), and advanced (G5), whereas intermediates (G3) also had significantly higher depression scores than those who rated themselves as advanced (G5). 

### 3.2. Validation of the Instruments

The current study also aimed to validate the instrument used to measure study abroad students’ coping strategies and ISE. For the scale validations, initial factor analysis was performed with SPSS version 26.0 (IBM, Armonk, NY, USA), whereas the succeeding confirmatory factor analysis was carried out using structural equation modeling with SPSS AMOS version 26.0 (IBM, Armonk, NY, USA) as part of a Hearne software lease.

For the coping strategies, correlations were first checked for the 35 items, with each having a value of not less than 0.30 between at least one other item, while not exceeding 0.85 [52]. Next, cross-loadings were examined with no lateral load of 0.32 or more [53]. The Kaiser–Meyer–Olkin (KMO) measure for the appropriateness of sampling was measured to be 0.87, which was well above the minimum limit of 0.50 [54]. Bartlett’s sphericity test was significant with *χ*^2^ (595) = 36,548.65, *p* < 0.001, signifying sampling adequacy [55]. Communalities were then calculated with all values above 0.40, confirming that the items had a common variance [56]. Following the initial analysis, a principal component analysis using varimax rotation was then performed to identify latent variables within the items [57]. Results showed that all of the items loaded successfully into seven subscales explaining 65.69% of the total variance.

Model fits for the confirmatory factor analysis were also assessed based on several criteria: the standardized root mean square residual (regardless of sample sizes [58], SRMR; values should be lower than 0.10 to indicate acceptable fit [59], but values less than 0.05 are much better [60]), a significant chi-squared test, root-mean-square error of approximation (RMSEA; values should be lower than 0.08 [61]), goodness-of-fit index (GFI), Tucker–Lewis Index (TLI), and comparative fit index (CFI), all of which should have values greater than 0.90 [62]. Finally, to compensate for the problems of multivariate normality, the bootstrap method (sampling repeated 2000 times) was used in the calculation [63].

Results exhibited a good model fit with SRMR = 0.043, CMIN (530) = 3210.72 with *p* < 0.001, RMSEA = 0.053 (90% CI 0.050 and 0.054), GFI = 0.91, TLI = 0.92, and CFI = 0.93, wherein each of the criteria fell within the prescribed cutoff values. In addition, a second-order confirmatory factor analysis was performed to validate the construct validity [64]. Results for the second-order confirmatory factor analysis also showed a good model fit with SRMR = 0.057, CMIN (544) = 3523.68 with *p* < 0.001, RMSEA = 0.054 (90% CI 0.052 and 0.056), GFI = 0.90, TLI = 0.91, and CFI = 0.92, wherein each of the criteria were also within the prescribed limits.

Furthermore, various criteria were also used to assess the construct validity and reliability of the instrument. For example, the composite reliability (CR; values should be greater than 0.50) [65], convergent validity (or average variance extracted, AVE; values should be greater than 0.50 [66] or above 0.40 is acceptable, if CR is greater than 0.60 [67]), discriminant validity (DV, calculated by taking the square root of AVE; values should be greater than their interconstruct correlations [66]), and the heterotrait:monotrait ratio of correlations (HTMT; values should be lower than 0.90 [68]) of the variables were calculated.

Table 2 shows the descriptive statistics, intercorrelations, reliabilities, and validities for the coping strategy subscales. Cronbach’s alpha reliability of the subscales ranges from 0.63 to 0.92, respectively, signifying sufficient to very good internal consistencies. The results indicated that CR are all greater than 0.50, acceptable AVEs of 0.40 and above, whereas DVs are higher than their interconstruct correlations (values in bold within the diagonal). HTMTs were all less than 0.90, all of which were within the cutoff parameters. After conducting the various stages of the factor analysis, the instrument for measuring study abroad coping strategies could be described as psychometrically sound [69].

For the ISE, similar criteria were used to assess the appropriateness of the 18 items. KMO was computed at 0.87, whereas Bartlett’s sphericity test was significant with *χ*^2^ (153) = 16,502.43, *p* < 0.001. Moreover, communalities were all above 0.40. A principal component analysis using the varimax rotation was then performed, which successfully loaded four subscales that explained 65.37% of the total variance. In addition, confirmatory factor analysis using structural equation modeling results showed a good model fit, with SRMR = 0.040, CMIN (125) = 1025.91 with *p* < 0.001, RMSEA = 0.062 (90% CI 0.059 and 0.066), GFI = 0.94, TLI = 0.93, and CFI = 0.95, wherein each of the criteria fell within the prescribed cutoff values. Importantly, a second-order confirmatory factor analysis also exhibited a good model fit with SRMR = 0.044, CMIN (127) = 1069.13 with *p* < 0.001, RMSEA = 0.063 (90% CI 0.060 and 0.067), GFI = 0.94, TLI = 0.93, and CFI = 0.94, wherein each of the criteria were also within the prescribed limits.

Table 3 shows the descriptive statistics, intercorrelations, reliabilities, and validities for the ISE subscales. Cronbach’s alpha reliability of the subscales ranged from 0.64 to 0.89, respectively, signifying sufficient to good internal consistencies. CR were all greater than 0.50, AVEs of 0.40 and above, whereas DVs were higher than their interconstruct correlations (values in bold within the diagonal). The HTMTs were all less than 0.90, all of which fell within the cutoff parameters. Having undergone the various stages of factor analysis, the instrument for measuring ISE is now considered to be psychometrically sound.

### 3.3. Regression Analysis

Hierarchical multiple regression analysis was performed to determine the role of coping strategies, ISE, and Mandarin Chinese and English language proficiency in predicting depression. Variables associated with depression were entered using a two-step procedure. First, in order to control for possible effects of demographic background, age (in years), gender (0 = female, 1 = male), study level (1 = undergraduate, 2 = master’s, and 3 = doctoral), length of stay (in months), whether their families were immigrants or not (0 = no, 1 = yes), and whether the students lived in Asia (0 = no, 1 = yes), were included as control variables in the equation. In the second step, the predictor variables of Mandarin Chinese and English language proficiency, coping strategies, and ISE were entered into the equation.

Table 4 displays the results of the hierarchical multiple regression analysis. For study-abroad-related depression, the control variables age (β = 0.091, *t* (1863) = 3.86, *p* < 0.001), immigrants (β = 0.058, *t* (1863) = 2.50, *p* < 0.05), and Asian (β = 0.054, *t* (1863) = 2.34, *p* < 0.05) all showed significant associations, and together explained 1.90% of the variance (*F* [6, 1863] = 5.97, *p* < 0.001). Furthermore, all of the predictors Chinese language (β = −0.050, *t* (1859) = −2.21, *p* < 0.05), English language (β = −0.058, *t* (1859) = −2.64, *p* < 0.01), coping strategies (β = −0.193, *t* (1859) = −7.40, *p* < 0.001), and ISE (β = −0.161, *t* (1859) = −6.22, *p* < 0.001) increased the explained variance to 12.50% (*F* [4, 1859] = 56.09, *p* < 0.001).

### 3.4. Moderation Analysis

Interaction! software, developed by Daniel Soper (https://www.danielsoper.com/Interaction/) and accessed on 25 December 2021, was used to assess the moderating effects of Mandarin Chinese and English language proficiency and ISE on the relationship between coping strategies and depression. In addition, to better understand the interactions within the moderation [70], simple slope comparisons between high (+2 SD) and low (−2 SD) values of the moderators were also performed using Interaction! software [71]. Simple slope difference tests are mostly used to determine three-way interactions within moderated multiple regression models [72]. More specifically, simple slope difference tests are used to test the impact of extreme values [73], high (+2 SD) predictors and their lower (−2 SD) counterparts on the relationship between coping strategies and depression. Notably, all variables and predictors were standardized and centered prior to calculation to make the results easier to interpret [74].

Table 5 and Table 6 shows the results of the moderation analysis for the effects of Mandarin Chinese and English language proficiency on the relationship between coping strategies and depression. As a result, neither interactions of Mandarin Chinese proficiency (β = −0.28, *t* (1860) = −0.92, *p* = 0.358) nor English proficiency (β = −0.12, *t* (1860) = −0.25, *p* = 0.800) have been able to establish a significant relationship between coping strategies and depression.

Table 7 shows the results of the moderation analysis for the effects of ISE on the relationship between coping strategies and depression. In addition to the previous six controlling variables, both Mandarin Chinese and English language proficiency were also treated as controlling variables. Results showed that the total model accounted for 12.70% (*F* [11, 1858] = 24.61, *p* < 0.001) of the variance in depression. The results also indicated that the control variables age (β = 0.11, *p* < 0.001), Mandarin Chinese proficiency (β = −0.34, *p* < 0.05), and English language (β = −0.60, *p* < 0.05) significantly predicted depression. In addition, coping strategies (β = −3.34, *p* < 0.001), ISE (β = −2.25, *p* < 0.001), and the interaction between coping strategies use and ISE (β = −1.23, *p* < 0.05) were all statistically significant in the model. The effect size of the interaction was considered small, with f^2^ = 0.14 [75].

**Figure 2:**
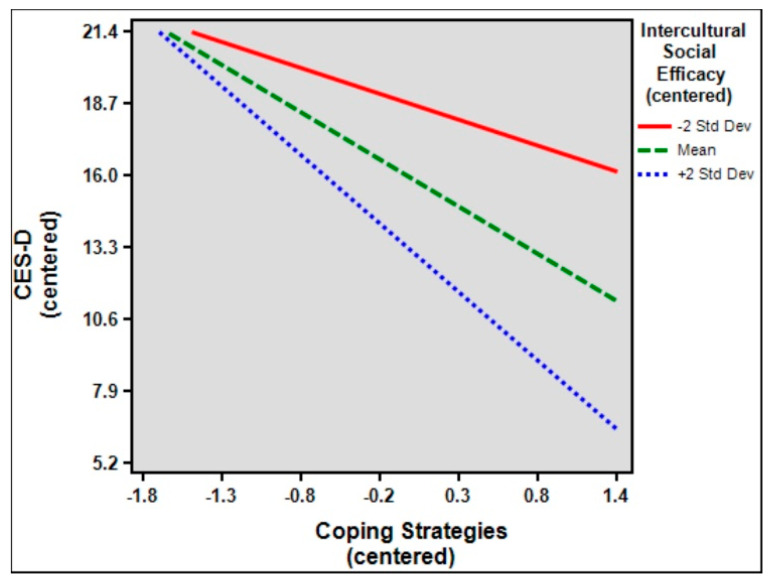
Simple slope plot for the moderation effect of ISE.

Table 8 shows the results of the simple slope models coping strategies, ISE, and depression. Results showed that the relationship between coping strategies and depression was significant among high (+2 SD with slope β = −4.85, *p* < 0.001) and low (−2 SD with slope β = −1.83, *p* < 0.001) ISE (β = −3.02, *p* < 0.001) [70]. Figure 2 shows the simple slope plot for the moderation effect of ISE, indicating that ISE strengthens the negative relationship between coping strategies and depression.

## 4. Discussions

In order to determine the primary objective of this study, several analyses were conducted. Participants were described along with the distribution of depression. As already mentioned, the participant population consisted of 925 female and 945 male students. The average age was 26 years old and the average length of stay was about 15 months. Upon further comparisons with the extent of depression, no significant differences were found in gender, study level, and length of stay. However, several significant differences in the group mean were found. In terms of age, the results showed that older participants were significantly more likely to have higher depression scores (but not to the point of depression). This finding is in line with an earlier study of American students in which younger students tend to have higher positive effect and less depression [76]. Nonetheless, this can be culture-dependent; in some Asian cultures, people may prefer not to interact with others and be alone, making them more likely to suffer from depression [77]. For the present study, older participants were most likely international students pursuing either their master’s or doctor’s degrees, and given the current competitive job market, these graduate students are highly likely to be stressed with regard to writing their dissertation and future career outlook. Some have also reported that the highly conflicting feelings that arise before leaving (a few months before returning home) are also a cause for stress and anxiety [78]. 

As expected, significant differences were found with participants who are immigrants. Participants who have an immigrant background have significantly higher degrees of depression as compared with the non-immigrants. Thus far, it has been assumed that participants with an immigrant background would tend to be more familiar and exposed to intercultural situations; hence, less depression. In reality, studies have found that many immigrants still suffer from identity issues or gaps that may actually contribute to depression [79]. Significant differences were also found among students who came from Asia with Asian students having significantly higher levels of depression than non-Asian. Findings confirmed that Asian students are likely to experience more academic pressure, resulting in greater stress than students from other parts of the world [38,39].

As for the issues of language and depression, both Mandarin Chinese and English language proficiency showed significant differences. Although there is no clear trend that depression is related to either higher or lower Mandarin Chinese proficiency, post hoc results merely indicated that participants with high intermediate levels in Mandarin Chinese tend to have a higher degree of depression as compared with the other language levels. It would be understandable that entry-level Mandarin Chinese participants would have higher levels of depression, concurring with previous studies on the necessity to master the language of the host country in order for study abroad to become fruitful and effective [80,81]. It should be noted, however, that when students reach a good enough level of language proficiency, their self-reported proficiency may become ambiguous [46].

In terms of English proficiency, it is quite evident that participants with less proficiency in English are more likely to experience depression; to the effect that participants who are new to English are actually depressed. This might also be due to the fact that more and more courses in Taiwan are shifting to English as a medium of instruction, although Mandarin Chinese is still the majority. Nonetheless, this is quite interesting, because Mandarin Chinese is supposedly more widely spoken in Taiwan, not English. However, as Yu et al.’s [17] framework suggested, bilingualism (particularly proficiency in English and the language of the host country) serves as an important agency for successful sociocultural adaptation.

Table 2 also shows significant intercorrelations between the subscales, meaning that these variables are related to each other; in other words, there is some degree of overlap between what the subscales are trying to measure [82]. In addition, the highest mean score within the subscales is self-actualization (M = 3.11, SD = 0.79), which suggests that participants are fairly confident about their talents and potentials, which is very encouraging. Self-actualization is closely related to self-efficacy, which is also a belief in one’s own abilities [83]. Indeed, it is suggested that study abroad experiences can create a context of empowerment, agency, and self-actualization [84]. For the current group of participants, higher self-actualization may refer to their feeling of confidence and competence. Furthermore, results also showed that health responsibility (M = 2.77, SD = 1.05) was the lowest-scoring subscale, meaning that participants were only moderately concerned about their health. This is a worrying issue, because international students ought to be aware of their health responsibilities by themselves. It is thus necessary for international students to receive timely reminders and appropriate training to minimize health risks [85,86]. 

Table 3 shows the significant intercorrelations between the ISE subscales, which also indicate that these variables are related to each other. In addition, the highest mean score within the subscales is the absence of social difficulties (M = 3.95, SD = 0.77), suggesting that participants are perfectly capable of dealing with social situations, such as interacting with new acquaintances, and finding their way around peers and teachers. According to Yu et al.’s framework, interactions and engagements with other individuals, whether in the classroom or in the community, are effective means for promoting sociocultural adaptation [8,17,19,24]. Hence, this finding is actually a good sign, suggesting that the current group of international students are most likely able to behave socially. Likewise, the results showed that friendship initiative (M = 3.05, SD = 1.01) was the lowest-scoring subscale, which indicated that participants lacked enthusiasm for initiating the first contact, although when the ice was already broken, communication should be seamless.

Hierarchical multiple regression analysis confirmed Yu et al.’s framework, wherein language (both Mandarin Chinese and English), coping strategies, and ISE were key factors in sociocultural adaptation, thus having the effect of lowering depression. These findings appear to support the role of language, coping strategies, and ISE in alleviating depression. As for the moderation analyses, neither Mandarin Chinese nor English language proficiency have been able to establish a significant interaction in the relationship between coping strategies and depression, suggesting that coping strategies are probably not language-dependent. Therefore, it can be presumed that both Mandarin Chinese and English language proficiency are not moderators; however, they can be considered as predictors of study-abroad-related depression as presented in the regression results. Initially, it was hypothesized that the language proficiency (Mandarin Chinese and English) acts as a moderator in the relationship between coping strategies and depression. The better one is at either of the two languages, the greater the impact it will have on coping strategies and depression. The results of this study, however, only showed that both languages were significant predictors (including coping strategies themselves), and which were noted to have the tendency of lowering depression levels. The moderation analysis showed that ISE is capable of moderating the relationship between coping strategies and depression. More specifically, ISE can help relieve depression. It is important that although both Mandarin Chinese and English language proficiency were not moderators, improved skills in either language should be able to help build the participants’ confidence towards better interactions [18]. Furthermore, increased exposure to a host country’s language also boosts cultural learning [10]. Nevertheless, language skills alone are not sufficient, but should be supplemented by culture-specific coping strategies [87].

## 5. Implications and Conclusions

### 5.1. Implications

This study provides a close-up view of depression among international students and argues for further investigation. First, descriptive, correlational, and group (independent samples *t*-test and analysis of variance) analysis showed that background demographic variables seemed to exert some influence on the level of depression. Participants that are older and have an immigrant background are more prone to depression; however, gender and length of stay appear to have no effect on depression, in stark contrast to previous studies [27,28]. Second, background demographic variables were controlled to determine the effects of language, coping strategies, and ISE on depression, which were confirmed and followed the study’s assumption, that knowledge of the host country’s language (for the current study, Mandarin Chinese) and English, coping strategies, and ISE are all able to reduce study-abroad-related depression. Third, moderating effects of both Mandarin Chinese and English language proficiency were not supported. However, ISE demonstrated significant interactions with coping strategies, suggesting that intercultural social efficacy does moderate the relationship between coping strategies and study-abroad-related depression. Lastly, comparison between the extreme ISE—high (+2 SD) and low (−2 SD)—indicated that a higher ISE tends to further increase the negative relationship between coping and depression (as represented by a steeper slope). In other words, increasing ISE will help improve coping strategies, which, in turn, will help reduce depression.

Discussions of implications would be incomplete without mention of the broader global structural context of international students in host nation higher education institutions. This means that contemporary elements of modern life could also influence the mental health situation among international students. For example, an expanding awareness of mental health literacy and media reporting on mental health and illness are developing on many social media platforms. Increasing mental health awareness through social media is a contemporary phenomenon because it reaches many people in a short time frame. This might include reports of increased rates of mental illness among the youth and expanded definitions and available resources for making sense of what constitutes mental health problems among international students in host nation institutions worldwide. At the same time, recent structural and cultural changes, along with growth in the global economy, have produced greater pressures and stress in the lives of all students.

Recent trends in higher education are also part of the broader structural context; the marketization of higher education and the construction of students as “consumers” creates new opportunities as well as new pressures. New individualized online learning environments, for example, are more complex and demanding than those of the past, when collective supports were more available. That said, these new learning environments may induce social isolation that can be addressed within institutions committed to ensuring good mental health conditions of international students.

This global snapshot suggests a mismatch between the broader structural context and the intricacies of micro-level practices and services. Although university support services fill a vacuum for mental health resources for all students, the difficulties faced by international students might be intensified. Despite the low levels of depression found in this study, preventative measures are still required to reduce the severity and risk of negative consequences associated with depression. This could include the provision of Mandarin Chinese language courses and English language courses for non-English speakers. International student offices could help organize activities where students can make new friends and help newcomers settle. Forming co-national clubs could also be beneficial to make students feel at home, hence promoting greater social interaction. However, co-national clubs can also act as a deterrent to further intercultural contacts or interactions and should therefore be treated with caution. This might be the case for international students who find themselves without traditional social markers with which to navigate experiences.

The current study has certain limitations which should be noted. The data analysis revealed only limited information about the students’ personal, situational, and contextual characteristics. In addition to the characteristics included in this study, there may be other factors which contribute to the challenges international students experience while studying abroad. For instance, these factors may include the students’ country of origin, their academic discipline and performance, their housing, and the location of the host institution. Future research could examine these variables from a narrative perspective to include student voice and identify the unmet needs of international students. This would benefit international students as well as higher education institutions and universities worldwide that are positioned in a competitive, market-driven environment.

### 5.2. Conclusions

In summary, study-abroad-related depression is a real phenomenon and not limited to students studying abroad in Taiwan. The most effective action universities can take to prevent this from happening is to identify students at a heightened risk for depression. Early intervention and prevention measures to avert mental health crises are tools all universities should earmark as a priority. Ongoing awareness to determine potential solutions should be included as an essential component of university mental health resources for all students.

## Figures and Tables

**Table 1:** Participants’ demographics and the distribution of depression.

Category	Groups	*n* (%)	CES-D (Mean ± SD)	*F*/*t*	*p*/Post Hoc
Age (years)				10.73	<0.001
	G1: ≤25	1213 (65%)	11.44 (8.95)		G2 > G1
	G2: 26 to 35	492 (26%)	13.30 (8.16)		G3 > G1
	G3: >35	165 (9%)	13.54 (7.19)		
Gender				1.83	0.067
	G1: Female	925 (49%)	11.74 (8.45)		
	G2: Male	945 (51%)	12.48 (8.83)		
Study level				1.23	0.294
	G1: Undergraduate	1160 (62%)	12.05 (8.09)		
	G2: Master’s	467 (25%)	11.86 (9.71)		
	G3: Doctoral	243 (13%)	12.90 (9.06)		
Length of stay (months)				2.00	0.113
	G1: ≤12	1352 (72%)	11.81 (8.09)		
	G2: 13 to 24	182 (10%)	13.08 (9.94)		
	G3: 25 to 36	123 (7%)	12.83 (10.19)		
	G4: >36	213 (11%)	12.77 (9.80)		
Immigrants				3.12	0.002
	G1: No	1545 (83%)	11.83 (8.65)		G2 > G1
	G2: Yes	325 (17%)	13.47 (8.52)		
Asian				2.21	0.027
	G1: No	482 (26%)	11.39 (8.12)		G2 > G1
	G2: Yes	1388 (74%)	12.36 (8.82)		
Mandarin Chinese Proficiency				6.17	<0.001
	G1: Beginner	168 (9%)	12.98 (8.76)		G4 > G5
	G2: Low intermediate	76 (4%)	11.32 (8.00)		
	G3: Intermediate	223 (12%)	12.49 (8.21)		
	G4: High intermediate	417 (22%)	13.63 (8.23)		
	G5: Advanced	986 (53%)	11.30 (8.86)		
English Proficiency				12.23	<0.001
	G1: Beginner	24 (1%)	19.00 (7.89)		G1 > G3, G4, G5
	G2: Low intermediate	58 (3%)	17.71 (8.04)		G2 > G3, G4, G5
	G3: Intermediate	171 (9%)	12.66 (7.56)		G3 > G5
	G4: High intermediate	913 (49%)	11.34 (8.58)		
	G5: Advanced	704 (38%)	12.29 (8.79)		

Notes. *N* = 1870. SD = standard deviation.

**Table 2:** Descriptive statistics, intercorrelations, reliabilities, and validities for the coping strategies.

Subscales	Alpha	M	SD	CR	AVE	SS	SA	CR	FS	HR	DR	SR
Social support (SS)	0.92	4.06	0.81	0.93	0.61	**0.78**	0.23	0.19	0.25	0.12	0.12	0.22
Self-actualization (SA)	0.91	4.11	0.79	0.91	0.63	0.21 **	**0.80**	0.29	0.46	0.08	0.21	0.46
Classroom rapport (CR)	0.88	3.73	0.78	0.88	0.59	0.17 **	0.26 **	**0.77**	0.21	0.05	0.20	0.27
Family support (FS)	0.88	3.88	0.94	0.88	0.59	0.23 **	0.41 **	0.19 **	**0.77**	0.12	0.15	0.25
Health responsibility (HR)	0.81	2.77	1.05	0.82	0.55	0.11 **	0.07 **	0.04 **	0.10 **	**0.74**	0.40	0.26
Daily routine (DR)	0.77	3.31	0.98	0.75	0.45	0.10 **	0.18 **	0.16 **	0.12 **	0.32 **	**0.67**	0.57
Self-relaxation (SR)	0.63	3.66	0.89	0.64	0.40	0.17 **	0.35 **	0.20 **	0.19 **	0.18 **	0.39 **	**0.61**

Notes. *N* = 1870. Overall Cronbach’s alpha reliability = 0.88. M = mean, SD = standard deviation, Alpha = reliability of subscale, CR = composite reliability, AVE = average variance extracted (convergent validity). Discriminant validities are in bold within the diagonal. Pearson’s correlations are below the diagonal, whereas heterotrait:monotrait ratio values of correlations are above the diagonal. ** *p* < 0.01.

**Table 3:** Descriptive statistics, intercorrelations, reliabilities, and validities for the ISE.

Subscales	Alpha	M	SD	CR	AVE	ASD	SC	SI	FI
Absence of social difficulty (ASD)	0.89	3.95	0.77	0.88	0.48	**0.69**	0.42	0.27	0.22
Social confidence (SC)	0.88	3.57	0.89	0.88	0.61	0.38 **	**0.78**	0.34	0.45
Showing interest (SI)	0.86	3.24	0.94	0.87	0.68	0.23 **	0.30 **	**0.83**	0.39
Friendship initiative (FI)	0.64	3.05	1.01	0.65	0.48	0.17 **	0.34 **	0.29 **	**0.69**

Notes. *N* = 1870. Overall Cronbach’s alpha reliability = 0.88. M = mean, SD = standard deviation, Alpha = reliability of subscale, CR = composite reliability, AVE = average variance extracted (convergent validity). Discriminant validities are in bold within the diagonal. Pearson’s correlations are below the diagonal, whereas heterotrait:monotrait ratios of correlations are above the diagonal. ** *p* < 0.01.

**Table 4:** Hierarchical multiple regression analysis of study-abroad-related depression.

	Predictors	*F* Change	*t*	df	B	SE	β	R^2^ Change	VIF
Dependent Variable: CES-D									
I.	Constant				7.287	0.994			
	Control variables	5.97 ***		6, 1863				0.019	
	Age		3.86 ***		0.117	0.030	0.091		1.06
	Gender		1.19		0.478	0.401	0.028		1.02
	Study level		1.01		0.304	0.302	0.025		1.18
	Length of stay (months)		0.82		0.008	0.010	0.021		1.21
	Immigrants		2.50 *		1.328	0.530	0.058		1.03
	Asian		2.34 *		1.070	0.458	0.054		1.02
II.	Predictors	56.09 ***		4, 1859				0.106	
	Chinese language		−2.21 *		−0.339	0.153	−0.050		1.07
	English language		−2.64 **		−0.615	0.233	−0.058		1.04
	Coping strategies		−7.40 ***		−3.359	0.454	−0.193		1.45
	ISE		−6.22 ***		−2.266	0.364	−0.161		1.43

Notes. *N* = 1870. df = degrees of freedom, B = unstandardized coefficients, SE = standard error, β = standardized coefficients, and VIF = variance inflation factor. ISE = intercultural social efficacy. Age is in years. Gender: 0 = female, 1 = male. Study level: 1 = undergraduate, 2 = master’s, 3 = doctoral. Length of stay is in months. Immigrants and Asian: 0 = no, 1 = yes. * *p* < 0.05, ** *p* < 0.01, *** *p* < 0.001.

**Table 5:** Moderation analysis for coping strategies, Mandarin Chinese proficiency, and depression.

Full Regression Model	β	SE	*t*	*p*	LLCI	ULCI
Predictor variables						
Constant	12.28	0.86	14.27	<0.001	10.589	13.965
Covariates						
Age	0.10	0.03	3.34	<0.001	0.041	0.158
Gender	0.39	0.38	1.02	0.308	−0.362	1.146
Study level	0.18	0.29	0.62	0.538	−0.390	0.747
Length of stay	0.01	0.01	1.40	0.162	−0.005	0.031
Immigrants	0.66	0.51	1.29	0.198	−0.344	1.660
Asian	0.90	0.44	2.04	0.041	0.035	1.755
Main effects						
Coping strategies	−3.88	1.28	−3.03	0.002	−6.388	−1.368
Mandarin Chinese proficiency	−0.35	0.15	−2.25	0.025	−0.653	−0.045
Two-way interaction						
Coping strategies X Mandarin Chinese	−0.28	0.30	−0.92	0.358	−0.867	0.313
Model fit	R^2^	Adjusted R^2^	f^2^			
	0.103	0.098	0.11			

Notes. All variables and predictors were standardized and centered prior to computing. *N* = 1870. β = standardized coefficients, SE = standard error, LLCI = lower-level confidence interval, and ULCI = upper-level confidence interval. Age is in years. Gender: 0 = female, 1 = male. Study level: 1 = undergraduate, 2 = master’s, 3 = doctoral. Length of stay is in months. Immigrants and Asian: 0 = no, 1 = yes.

**Table 6:** Moderation analysis for coping strategies, English proficiency, and depression.

Full Regression Model	β	SE	*t*	*p*	LLCI	ULCI
Predictor variables						
Constant	13.96	1.21	11.58	<0.001	11.597	16.324
Covariates						
Age	0.12	0.03	4.26	<0.001	0.067	0.181
Gender	0.34	0.38	0.88	0.377	−0.414	1.094
Study level	0.10	0.29	0.34	0.737	−0.470	0.664
Length of stay	0.01	0.01	1.35	0.177	−0.006	0.030
Immigrants	0.66	0.51	1.30	0.195	−0.339	1.663
Asian	0.84	0.44	1.93	0.054	−0.015	1.705
Main effects						
Coping strategies	−4.36	2.01	−2.17	0.030	−8.304	−0.422
English proficiency	−0.69	0.24	−2.92	0.003	−1.159	−0.229
Two-way interaction						
Coping strategies X English proficiency	−0.12	0.47	−0.25	0.800	−1.035	0.798
Model fit	R^2^	Adjusted R^2^	f^2^			
	0.104	0.100	0.12			

Notes. All variables and predictors were standardized and centered prior to computing. *N* = 1870. β = standardized coefficients, SE = standard error, LLCI = lower-level confidence interval, and ULCI = upper-level confidence interval. Age is in years. Gender: 0 = female, 1 = male. Study level: 1 = undergraduate, 2 = master’s, 3 = doctoral. Length of stay is in months. Immigrants and Asian: 0 = no, 1 = yes.

**Table 7:** Moderation analysis for coping strategies, ISE, and depression.

Full Regression Model	β	SE	*t*	*p*	LLCI	ULCI
Predictor variables						
Constant	15.04	1.31	11.46	<0.001	12.466	17.614
Covariates						
Age	0.11	0.03	3.73	<0.001	0.052	0.168
Gender	0.44	0.38	1.16	0.248	−0.306	1.185
Study level	0.15	0.29	0.52	0.605	−0.413	0.710
Length of stay	0.01	0.01	1.41	0.160	−0.005	0.031
Immigrants	0.75	0.50	1.49	0.137	−0.238	1.740
Asian	0.81	0.43	1.87	0.061	−0.038	1.660
Mandarin Chinese proficiency	−0.34	0.15	−2.25	0.025	−0.644	−0.044
English proficiency	−0.60	0.23	−2.58	0.010	−1.055	−0.143
Main effects						
Coping strategies	−3.34	0.45	−7.36	<0.001	−4.227	−2.448
ISE	−2.25	0.36	−6.19	<0.001	−2.966	−1.539
Two-way interaction						
Coping strategies X ISE	−1.23	0.52	−2.38	0.018	−2.237	−0.215
Model fit	R^2^	Adjusted R^2^	f^2^			
	0.127	0.122	0.15			

Notes. All variables and predictors were standardized and centered prior to computing. *N* = 1870. β = standardized coefficients, SE = standard error, LLCI = lower-level confidence interval, and ULCI = upper-level confidence interval. Age is in years. Gender: 0 = female, 1 = male. Study level: 1 = undergraduate, 2 = master’s, 3 = doctoral. Length of stay is in months. Immigrants and Asian: 0 = no, 1 = yes.

**Table 8:** Simple slope models for coping strategies, ISE, and depression.

Simple Slope Models	β	SE	*t*	*p*	LLCI	ULCI
Groupings						
+2 SD (*n* = 45)						
Intercept	12.26	0.46	−10.63	<0.001	−5.743	−3.954
Slope	−4.85					
Mean (*n* = 1780)						
Intercept	15.04	0.45	−7.36	<0.001	−4.227	−2.448
Slope	−3.34					
−2 SD (*n* = 45)						
Intercept	17.82	0.45	−4.02	<0.001	−2.717	−0.937
Slope	−1.83					
Simple slopes difference (+2 SD, −2 SD)						
	−3.02	0.23	−12.99	<0.001		

Notes. All variables and predictors were standardized and centered prior to computing. *N* = 1870. β = standardized coefficients, SE = standard error, LLCI = lower-level confidence interval, and ULCI = upper-level confidence interval.

## Data Availability

Data for the current study are available upon written request to the corresponding author.
